# Sex-Specific Thresholds for Cardiac Biomarkers—We Need to Move Forward

**DOI:** 10.31083/j.rcm2403086

**Published:** 2023-03-08

**Authors:** Ronstan Lobo, Allan S. Jaffe

**Affiliations:** ^1^Department of Cardiovascular Medicine, Mayo Clinic, Rochester, MN 55905, USA; ^2^Department of Laboratory Medicine and Pathology, Mayo Clinic, Rochester, MN 55905, USA

**Keywords:** sex, troponin, natriuretic peptides, ST2, galectin

## Abstract

Cardiovascular biomarkers play a major diagnostic role for cardiologists. Different biomarkers provide different insights into a variety of cardiovascular conditions and in doing so they improve diagnosis and management. Often, these biomarkers are deployed without carefully evaluating the use of sex-specific cut off values. It is now becoming apparent that the use of such cut off values can improve prognostication and discrimination in some clinical situations. This review paper will focus on the data indicating that there is benefit to the use of sex-specific thresholds. It should be clear that these thresholds will vary depending on the analyte being measured and the specific clinical indication for which the patients are being evaluated; and sex-specific cut off values may be important in some situations but not others. Nonetheless, it is now clear that when evaluating sex-specific cut off values, one often finds benefit. We will highlight these situations using specific cardiac biomarkers as examples.

## 1. Introduction

Cardiovascular biomarkers play an important role in the diagnosis, management, and risk stratification of patients with cardiovascular disease. It is important that these markers are deployed optimally for each unique population of patients in the proper clinical setting. With the increasing sensitivity of new cardiovascular biomarkers, differences between different populations are now apparent including differences related to age, race, and sex [1, 2]. These differences warrant not only understanding but studies defining their optimal clinical use. There is also a need to understand why cardiovascular disease (CVD) continues to kill more females than males annually in the United States [3]. Concerns have been raised of the possibility that cardiovascular disease is recognized more readily in males. This has been called “a diagnosis gap” by some [4]. A potential example of this is in patients with ischemic heart disease (IHD) where reduced recognition due to the lack of sex-specific criteria could potentially account for the increased mortality observed in females [4]. There is acknowledgement that some of this is due to a lack of recognition, but some may be due to other contemporary social issues related to diversity and inclusion which are beyond the scope of this review [5]. In this review, we will focus on sex-specific differences in commonly used cardiac biomarkers and make the argument, using selected biomarkers and disease entities as examples, for the need of these biomarkers to be implemented based on sex-specific thresholds. It is likely in our opinion that in these situations, it would positively impact patient care.

## 2. Biomarkers of Myocardial Injury

Sex-specific cut off values for cardiovascular biomarkers have been advocate for years [6]. In 2007, the National Academy of Clinical Biochemistry (NACB) and the International Federation of Clinical Chemistry (IFCC) committee recommended the use of sex-specific reference limits for creatine kinase MB-isotype (CK-MB) and myoglobin measurements as in the past [7]. These recommendations have been extended to cardiac troponin (both conventional and high-sensitive assays) [8] and have been embraced by the 4th Universal Definition of Myocardial Infarction [9].

We have previously highlighted the importance of using sex-specific cutoffs for cardiac troponins [10]. Males have higher high-sensitivity cardiac troponin (hs-cTn) concentrations than females [11], likely related to the intrinsic differences between the sexes, although the exact mechanisms are not entirely clear. There also are differences in coronary pathophysiology. Males have a higher incidence of subclinical coronary artery disease (CAD) [12]. It is also known that the degree of atherosclerosis, left ventricular (LV) hypertrophy and cardiomyocyte apoptosis tends to be less in females [12], which may be related to the attenuating properties of estrogens on thrombus formation, vasoreactivity and vascular apoptosis [13]. Moreover, females also have smaller epicardial coronary arteries even after adjusting for age, body habitus and left ventricular mass [14]. These factors together with a higher myocardial blood flow in females even when corrected for body surface area, LV volume and hematocrit are associated with a significant increase in endothelial shear stress [13, 15]. Increases in sheer stress are associated with less focal lipid accumulation, less pathologic remodeling, and lower plaque instability [13]. Accordingly, it is not surprising that the mechanisms that lead to acute events are different. In males, plaque rupture is the primary cause of myocardial infarction (MI), but plaque erosion is more often the cause of coronary thrombosis in females, particularly in the premenopausal years [13]. Furthermore, females more often present with chest pain related to microvascular and endothelial dysfunction and/or diffuse coronary atherosclerosis, rather than a defined focal culprit plaque. These factors may be why there are fewer marked hs-cTn elevations in females than in males with acute ischemic events [16].

Biomarkers of myocardial injury such as hs-cTn are used in 2 contemporary settings: (a) as a prognostic marker in patients with stable cardiovascular disease; and (b) as a marker of acute coronary syndrome (ACS). Most of the focus has usually been on the acute situation as the indications for the use of hs-cTn as a prognostic marker though robust has lagged substantially behind the use of the marker in the acute setting. The situation is complex in patients with ACS. Some studies have shown no benefit of using sex-specific cutoffs in terms of predicting major cardiovascular outcomes [17]. However, many of these studies have only included patients with chest pain that raised suspicion for ACS. This may seem like an appropriate inclusion criterion, but these criteria can result in patients with atypical presentations being excluded. This may particularly affect females and especially those who are elderly (more of whom are females than males) who tend to present with both less classical chest discomfort and also atypical symptoms [16]. We have recently shown that in the U.S. where hs-cTn testing is used more extensively that there are increases not only in the diagnosis of myocardial injury but also in the diagnosis of MI in females with the use of sex-specific cut off values [18]. When a more inclusive approach of recruiting patients with ACS is implemented, more similar to that in the U.S. such as was done in the High-Sensitivity Troponin in the Evaluation of patients with suspected Acute Coronary Syndrome (High-STEACS) [19], sex-specific cut off values for high-sensitivity cardiac troponin I (hs-cTnI) resulted in a sizeable reclassification of myocardial injury (17%) with a twofold frequency in females compared to males. Despite this however, there were no significant differences in the primary outcome of recurrent MI or cardiovascular death at 1 year among reclassified patients (males or females) [19]. Some might be tempted to conclude that since the primary outcome did not change, sex-specific cutoffs might not be helpful [19]. However, there are 2 issues to consider. The first is that there is vast literature describing increases in hs-cTn in a variety of conditions, including uncontrolled hypertension, heart failure, tachycardia, anemia, carbon monoxide intoxication and a long list recently reviewed [9]. Although it is clear that increases in hs-cTn foreshadow worse outcomes, it is important to evaluate patients to define the cause before starting treatment. Unfortunately, both evaluations and treatments are often lacking in those without ischemic heart disease as in the High-STEACS study referred to above. This was documented clearly in their subsequent analysis [20] where they showed that despite the increases of type 1 MI, type 2 MI, and myocardial injury (raised hs-cTnI without evidence of ischemia), females were less likely to undergo evaluation for ischemia or receive antiplatelet therapy or secondary prevention strategies. While there was no overall change in the primary outcome (recurrent MI or cardiovascular death at 1 year) between females and males, when the Type 1 MI cases were looked at individually, females were still less likely to undergo coronary angiography (52% vs 73%) and the primary outcome in males decreased by 3% (18% to 15%) while the females had a higher rate of the primary outcome and this remained unchanged (19%) [20]. This stresses not only of the importance of sex-specific cutoffs, but also on acting on the results provided by the use of hs-cTn. The improvement in diagnostic accuracy alone cannot improve outcomes unless it changes the undertreatment of females and improves their management [5].

There are also abundant data showing that sex-specific cutoffs improve prognostication in patients with stable CAD and in primary prevention. In patients with stable coronary artery disease undergoing elective percutaneous coronary intervention, pre-procedural high-sensitivity cardiac troponin T (hs-cTnT) with the use of sex-specific cutoffs was proven to be a strong predictor of mortality in both males and females [21]. In an ancillary study of the BARI 2D trial [22], which evaluated a population of patients with diabetes and stable ischemic heart disease, lower concentrations of hs-cTnT observed in females were associated with an elevated risk of major cardiovascular (CV) events and death compared with males with similar hs-cTnT levels. Based on these data, the authors suggested that sex-specific thresholds for hs-cTnT, with lower thresholds for females were appropriate to identify those at elevated risk of major cardiovascular events [22]. A large scale prospective, population-based cohort study in Norway also showed hs-cTnI to be a significant predictor of cardiovascular death; this finding was more prominent in females than in males [hazard ratio (HR) 1.44 (1.31–1.58) vs 1.10 (1.00–1.20); *p* interaction < 0.001] [23]. These data suggest that there are different trajectories for males and females with chronic cardiovascular disease suggesting that using their unique values improves prognostication.

It is conceivable that some hs-cTn assays will have 99th percentile upper reference limits (URLs) that are very close to each other for males and females which may make it hard to show differences between the sexes. This may be the case with the hs-cTnI assay from Beckman-Coulter [11]. However, even then, getting used to the deployment of sex-specific cut off values would be advised as although the differences may not be present in the ACS patients, they will nonetheless likely persist in those who require cardiovascular risk stratification. What is clear is that the more one looks, the stronger the data become.

## 3. Biomarkers of Heart Failure

Although the overall lifetime risk of heart failure (HF) is similar between the sexes [24, 25], sex differences become apparent when the type of HF is considered. The lifetime risk of heart failure with reduced ejection fraction (HFrEF) is higher in males [26, 27]. The epidemiologia da insuficiência cardiaca e aprendizagem (EPICA) study [28] however showed the prevalence of heart failure of heart failure with preserved ejection fraction (HFpEF) was higher in females than in males. Similarly, a Mayo Clinic community study [29] showed the proportion of HFpEF (relative to HFrEF) increasing over time with females outnumbering males by about 2:1 among patients with incident HFpEF.

Some of the differences noted are related to differences in the pathophysiology of heart failure between males and females. Females with HFpEF were more likely to develop concentric LV remodelling, more severe diastolic dysfunction and higher LV filling volumes compared to males with HFpEF [30]. It appears that females develop thicker walls and thus smaller LV chambers during the remodelling process [31]. This increase in LV stiffness happens even though females are reported to have a lower tendency to develop myocardial fibrosis than males [32, 33]. This may partially be due to greater induction of renin-angiotensin-system (RAS)-related genes in the male compared to the female myocardium [34], or it may be due to dampening of the RAS by estrogens in females [35].

The higher risk of HFrEF in males compared to females may be attributable to the higher predisposition to macrovascular coronary artery disease and myocardial infarction, which is a major precursor to the development of HFrEF [36]. Coronary microvascular dysfunction on the other hand, is thought to play a major role in HFpEF [37]. The prevalence of microvascular dysfunction in heart failure with preserved ejection fraction (PROMIS-HFpEF) [38] study showed a strong association between coronary microvascular dysfunction in patients with HFpEF who either did not have large vessel CAD or who had been revascularized. Coronary microvascular dysfunction was present in 75% of patients with HFpEF and related to the severity of HF (as measured by N-Terminal prohormone of B-type natriuretic peptide [NT-proBNP]) and cardiac dysfunction (ventricular/atrial strain) as well as markers of systemic endothelial dysfunction [38]. As alluded to above on the role of microvascular dysfunction in the pathophysiology of coronary events in females, microvascular dysfunction also appears to play an accentuated role in the development of heart failure in females.

### 3.1 Natriuretic Peptides

Natriuretic peptides (NP) are a class of hormones secreted in response to hemodynamic stress. In Cardiology, there is a focus on those that are released in response to stretch and promote natriuresis, diuresis and reduction in vascular resistance as at least part of their mechanisms [39]. Natriuretic peptides are established markers for the diagnosis and prognosis of heart failure. Just like hs-cTn, it is also a useful biomarker for risk stratification in other cardiovascular disorders [40, 41]. A-type natriuretic peptide (ANP) and B-type natriuretic peptide (BNP) are primarily found in the myocardium. Significant clinical data are also available for NT-proBNP, which is the remaining 76-amino acid cleavage product of proBNP. It has a longer half-life than BNP. BNP or NT-proBNP are recommended by both the 2021 ESC [42] and 2022 AHA/ACC/HFSA [43] heart failure guidelines as diagnostic tests to support the diagnosis or exclusion of heart failure. However, neither of these guidelines recommend the implementation of sex-specific cutoffs [42, 43].

This may be surprising because it has been shown that both BNP and NT-proBNP are higher in females than in males [44, 45]. The reason for this is not totally well established. Sex hormones have been thought to play a role. Testosterone lowers cardiac natriuretic peptide levels via upregulation of neprilysin activity [46, 47]. There has also been evidence that postmenopausal hormone replacement therapy results in increased BNP concentrations [44, 48]. It is also known that obesity is associated with lower BNP levels because of greater clearance of the peptide from increased expression of the natriuretic peptide clearance receptor on adipose tissue, leading to its internalization and degradation [49]. However, there is also evidence to show that while obesity does lead to lower BNP levels, the difference in fat distribution between males and females might also play a role [50].

Given that there are significant differences in the normal ranges, one might expect different critical cut off values to be used clinically depending on the clinical condition being studied. However, this is not always the case. This reflects the fact that as physiological activators, NPs have a broad dynamic range such that marked increases are required to distinguish between a physiological response and a pathological one. For example, Wu *et al*. [51] reported that one requires a change of 90–130% to be sure one has not exceeded conjoint biological and analytical variation. These are also the levels reported in studies evaluating the change in NPs necessary to see differences in prognosis [52]. Large clinical trials and consensus documents have suggested that changes as low as 30% may be of significance [53, 54]. It may well be that is correct because those patients whose values are declining over a shorter time period continue to do so, thus correlating with outcomes. Thus, at present there are no data to support the use of sex-specific cut off values in patients with heart failure.

This may change however with time. Recent data suggest that values of NPs in the upper range of the putative normal reference values can be used to identify those with an increased propensity to the development of heart failure. The St Vincent’s Screening to Prevent Heart Failure (STOP-HF) trial [55] probed the use of a BNP value of 50 pg/mL or higher as a cut off to identify patients at risk of heart failure. Patients with BNP values ≥50 pg/mL were randomized to either a program of echocardiographic screening and collaborative care between the primary care physician and a cardiovascular specialist, or usual care. Despite this low cut off level, the intervention group had significantly lower rates of emergency hospitalization for major cardiovascular events (incidence rate ratio: 0.60; 95% CI: 0.45–0.81, *p* = 0.002) over time [55], suggesting that such low values might be valuable to identify those at risk for heart failure. One could even suggest that the advantage of this approach is that it included more females. Another similar example was the NT-proBNP selected prevention of cardiac events in a population of diabetic patients without a history of cardiac disease (PONTIAC) trial [56] which randomized diabetic patients with a NT-proBNP cut off of >125 pg/mL who were free of cardiac disease into either a control group or an intensified treatment group with renin-angiotensin system antagonists and beta blockers. The intensified treatment group had a significant reduction of the primary endpoint of hospitalization or cardiac death (hazard ratio: 0.351; 95% CI: 0.127–0.975, *p* = 0.044) in only 2 years [56]. The modest differences in normal values between the sexes may be much more prone to play a role in these sorts of studies than in those of more overt heart failure. It has also been shown that natriuretic peptide levels are much lower in patients with HFpEF than in patients with HFrEF [57, 58, 59]. With the higher incidence of HFpEF in females, sex-specific cut off values might also play a role in screening for heart failure [44]. Unless we look, we are unlikely to find differences.

### 3.2 Cardiac Troponin

In the acute heart failure setting, hs-cTn values are known to be increased significantly in most patients and increases are predictive of adverse outcomes [60, 61]. This is often also the case in more chronic disease [62]. Increases are associated with more severe disease and presage an adverse prognosis. To the best of our knowledge, no one has probed the use of sex-specific cut off values in this situation.

However, there are data using hs-cTn to predict development of incident HF. Data from the Mayo Clinic have shown that using the 80% percentile of sex-specific cut values for hs-cTnI and NT-proBNP allowed the robust prediction of HF in a primary prevention cohort from Olmsted County [63]. In a large meta-analysis involving nearly 67,000 patients, it was demonstrated that there is a strong association between hs-cTn and the development of incident HF in both males and females [64]. For incident heart failure, Yan and colleagues [65] published large study from a general population that showed hs-cTnI levels were independently associated with incident heart failure and different hs-cTnI cutoff values of 2.6 ng/L for females and 4.2 ng/L for males were derived to optimally identify individuals at risk. This comports to the fact that hs-cTn values in females are invariably lower than those in males. Thus, one would expect this phenomenon to also play out for the prediction of HF. In a population of stable chronic HF patients, a recent study showed improved prediction of the composite outcome of 1 year CV death and HF hospitalization when hs-cTnT values of 22 ng/L were used for females and 25 ng/L used for males [66]. These data are in keeping with most of the data in the primary prevention setting [67, 68].

## 4. Biomarkers of Inflammation

It is well established that inflammation contributes to the development of CVD [69, 70]. C-reactive protein (CRP) is an acute phase reactant released from the liver in response to cytokine stimulation, and particularly in response to interleukin (IL)-6 [71]. The development of the high-sensitivity CRP (hs-CRP) assay has allowed for the measurement of low levels of inflammation and has been used as a CVD risk marker in both males and females [72]. It has been shown that females have hs-CRP levels that are 30–50% higher than males, even after adjustment for CVD risk factors [73, 74, 75]. There are potentially a number of reasons for this, but it has been thought to be related to the difference in the genetic and hormonal make up of males and females [76]. This is also thought to perhaps contribute to the higher incidence of autoimmune disease in females [77]. This sex-based difference is not only seen in CRP but also other markers that change in response to inflammation including d-dimer, IL-18 and lipoprotein phospholipase A2 [1]. Adipose tissue contributes to overexpression of CRP, and has been strongly correlated with CRP levels, particularly among females [70]. However, other studies have found significant sex differences in CRP levels independent of obesity and age [2, 78].

Much of the research done with hs-CRP is in the field of prediction of long-term CVD risk. The joint AHA/CDC guidelines [79] have proposed cutoff points for CRP with low levels being <1 mg/L, moderate levels being 1–2.9 mg/L and high levels ≥3 mg/L. There were no sex-specific cutoffs advocated. A large meta-analysis looking at a number of these studies was published by Danesh *et al*. [80]. The overall population from the studies was male dominated (72% of subjects), and although they did analyze the data according to cutoffs based on the tertiles, they did not stratify by sex. This can be important as was shown in a study by Opotowsky *et al*. [81] which evaluated the prediction of clinical events in patients with congenital heart disease using hs-CRP. They found a significant relationship in both sexes, but it was weaker for females (HR = 2.19, 95% CI: 1.26–3.81, *p *< 0.001) than for males (HR = 4.72, 95% CI: 2.88–7.75, *p *< 0.001) [81], which is unsurprising given that females have higher baseline CRP levels. However, when they divided the results into sex-specific quartiles, there remained a significant relationship but there was a mild adjustment down in the hazard ratio for males (HR = 4.02, 95% CI: 2.45–6.60, *p *< 0.001) and a mild adjustment up in females (HR = 2.28, 95% CI: 1.30–4.01, *p* = 0.004) [81]. Other studies that have also implemented sex-specific quartiles have found significant associations between hs-CRP in both males and females with development of atrial fibrillation [82] and development of ischemic stroke and TIA [83]. The differences however have been small and may not be necessary in routine clinical practice.

## 5. Biomarkers of Fibrosis

### 5.1 Soluble Interleukin-1 Receptor-Like 1 (sST2)

ST2 is a member of the IL-1 receptor family and is a marker of cardiomyocyte stress and fibrosis. It has a transmembrane (ST2L) and soluble (sST2) isoform. The circulating form is believed to function as a “decoy” receptor for IL-33. IL33 is anti-fibrotic and anti-hypertrophic. By binding IL33, sST2 inhibits the effects of IL-33/ST2L signaling, removing its beneficial effects [84]. This results in adverse remodeling of the ventricular myocardium with myocyte hypertrophy, fibrosis, and a decline in function [85]. Therefore, sST2, though not nearly as robust a diagnostic biomarker as natriuretic peptides, has shown greater prognostic impact in all comers with dyspnea, perhaps because in addition to its effects on fibrosis and hypertrophy, it is also sensitive in detecting inflammation [84].

There is evidence for sex-specific differences in sST2 normal reference values, with higher sST2 concentration in males compared to females [86, 87]. The reasons for this remain unclear. Part of the reason may relate to the role of sex hormones. In the Framingham study, females on exogenous estrogen therapy had the lowest sST2 values [88]. However, a different study did not show that sST2 levels were associated with androgen or estrogen status [89]. Despite the clear difference in reference values between the sexes, most studies have used a single cut-off point of 35 ng/mL. This is primarily due to the use of the only US Food and Drug Administration (FDA)-approved Presage ST2 assay (Critical Diagnostics, San Diego, CA, USA), which has an FDA-approved prognostic cutpoint of 35 ng/mL [90].

Previously prognostic studies have shown elevated sST2 levels to be predictive of incident HF, overall and cardiovascular death [91, 92, 93]. Because of its prognostic value, it has become a part of the risk stratification strategy in HF guidelines in the United States but has not received the same strong recommendations as the natriuretic peptides and hs-cTn [94, 95]. However, it has not been included in the most recent 2022 American Heart Failure guidelines [43]. The use of sex-specific cutoffs has not been advised.

However, the usefulness of sex-specific cutoffs for sST2 was probed in a study done by Harmon *et al*. [96]. This study evaluated a large North American community-based cohort of asymptomatic (Stage A/B) HF subjects. Sex-specific cutoff values were obtained by stratifying results by sex and placing them into quartiles based on the distribution of the sST2 values [96]. When non-sex-specific univariate analysis was performed, sST2 was associated with HF, major adverse cardiovascular outcomes (MACE) and mortality but the association was significantly weaker following adjustment for cardiovascular risk factors and other cardiovascular biomarkers (NT-proBNP and hs-cTnI) [96]. However, when the sex-specific cutoffs were used, the univariate analysis continued to show significant associations with the aforementioned outcomes and remained strongly predictive despite adjusting for cardiovascular risk factors and other cardiovascular biomarkers [96]. Another more recent study involving 4540 patients found that using sex-specific cutoffs based on the population studied provided improved risk prediction compared to the use of previously standardized prognostic cutoffs [66]. It may well be that the enthusiasm for the use of sST2 for risk stratification has been blunted by the lack of use of sex-specific cut off values. That hopefully can be remediated by these more recent evaluations.

### 5.2 Galectin-3

Galectin-3 (Gal-3) is a lectin that is secreted by various immune cells, including mast cells, histocytes and macrophages [97]. It is readily secreted into biological fluids from injured cells and inflammatory cells and is thought to play an important role in the recruitment of macrophages to initiate the development of cardiac fibrosis and for that reason, it may identify those at risk for HF [97]. However, the analyte is not specific for the heart in that liver and/or renal fibrosis can also cause increases to be seen in the blood [98, 99].

Gal-3 levels have been associated with fat mass [100, 101], diabetes and chronic kidney disease [101, 102]. Gal-3 levels have been associated with adverse outcomes during follow-up in patients with acute and chronic HF [103, 104]. It has also been shown to be predictive of incident HF and mortality in general population studies [105, 106]. Similar to sST2, Gal-3 is recommended for risk stratification in HF guidelines in the United States [94, 95]. Once again, use of sex-specific cutoffs has not been advised.

However, population-based studies reveal that Gal-3 levels tend to be higher in females than in males [102, 105, 106]. The reason for this could be related to the higher fat mass in females for the same given body mass index (BMI) compared to males. Another potential cause is the difference in the comorbidity profile modulating expression of Gal-3 in females preferentially compared with males as was shown in the study by Lau *et al*. [107]. Could it be that probing sex-specific cut off values would be of value?

## 6. Limitations and Future Opportunities in Advancing the Field

The future is now. It is now clear that females with cardiovascular disease have different presentations, differences in the biology and physiology that lead to more overt disease and thus, it would not be surprising for them to have different baseline concentrations of a variety of biological markers. There was a lack of scrutiny of this issue in studies of ischemic heart disease [5]. This is unfortunate and we should guard against that occurring in other cardiac disease fields where biomarkers are used. We have summarized some of the sex-specific differences in the biomarkers we have discussed in Table 1 (Ref. [2, 74, 96, 102, 108, 109]).

**Table 1.** **Examples of Sex-specific cut off /median values**.

Test	Disease studied	Assay	Overall Cutoff/Median Value	Sex-Specific Cutoffs/Median Values		Reference
				Males	Females	
High-sensitivity Cardiac Troponin T	Myocardial infarction	Roche cTnT Gen 5 STAT	14 ng/L	17 ng/L	9 ng/L	[108]
High-sensitivity Cardiac Troponin I	Myocardial infarction	Abbott Architect STAT i	28 ng/L	35 ng/L	17 ng/L	[109]
NT-proBNP*	Heart Failure	Roche Elecsys proBNP II	196 ng/L	169 ng/L	254 ng/L	[2]
High-sensitivity CRP	Healthy Population	Roche/Hitachi 912 System, Tina-quant assay	20 mg/L	1.8 mg/L	3.3 mg/L	[74]
sST2	Heart Failure	Critical Diagnostics Presage ST2 assay	25.9 ng/mL	33.5 ng/mL	25.9 ng/mL	[96]
Galectin-3	Healthy Population	Alere Galectin-3	14.8 ng/mL	13.7 ng/mL	15.3 ng/mL	[102]

*The authors acknowledge that the normal values though always different between males and females change with increasing age as well.

There are limitations that should be addressed before the use of sex-specific cutoffs can be implemented. Firstly, sex-specific differences of a specific analyte can only be probed if the assay is sensitive enough to not only detect differences in the reference “healthy” population, but also within each sex strata in the healthy population. It is also important that sex-specific analysis be done in the context of the specific disease being studied. For example, cutoffs used with hs-cTn is assessing for acute myocardial infarction may be very different from accessing myocardial injury from other causes, and thus, the appropriate analyses would need to be performed and the study populations need to include an adequate number of females, particularly older females. Until this is done, it will continue to be difficult to establish accurate sex-specific cutoffs.

Our default position when developing new biomarkers should be to always probe sex-specific cut off values to see if the approach will improve the identification of females with disease. Some suggestions to achieve this are listed below.

### 6.1 Appropriate Selection of a Reference “Healthy” Population

In assessing the utility of sex (as well as age and race) specific cutoffs, one has to consider the importance of the reference “healthy” population that is used to determine the reference limit/interval for the various groups. It is important to ensure that the “healthy” population that is used is truly free of subclinical disease. Various methodologies are used to assess this, including history taking, measurement of biomarkers and imaging. If there is underlying subclinical disease, these will cause differences in measured values between the sexes and age groups. For example, Koerbin *et al*. [110] showed that there were clinically significant male-female differences in the hs-cTnI 99th percentile values. However, after they ‘coned’ the results by progressively excluding patients with abnormal renal function, elevated NT-pro BNP, clinical history, and abnormal echo, they found that only patients <55 years showed a marked sex difference in the 99th percentile. This would suggest that even in subjects that are apparently healthy, simple coning of subjects using parameters that indicate underlying subclinical disease can result in changes to the 99th percentile value, with particular reductions noted in males [110]. These findings were also noted in other studies [111, 112, 113], highlighting the importance of evaluating reference populations carefully as minor changes that result from subclinical disease can distort the sex-specific cut off values. To ensure consistency between groups, we endorse the use of separate cohorts of at least 400 males and 400 females in all reference range studies as recently proposed by the IFCC educational task force [114].

### 6.2 Determine Prognostic Cut-Offs for a Specific Clinical Disease, Stratified by Sex

When there are differences in reference ranges, probing the data to optimize sex-specific prognostic values is obligatory. However, even if that is not the case, sex-stratified analysis should be performed in order to assess for sex-specific differences; statistical modeling with adjustment for sex alone is inadequate. There is increasing literature to help guide investigators in this matter [115, 116].

## 7. Conclusions

We have elaborated above the data that supports the use of sex-specific cutoff values for a variety of analytes that are used in cardiology. Previously, these differences have rarely been probed so it is impossible in any specific situation to define why the impact was missed. Now, when these cut offs are deployed for biomarkers such as troponin, natriuretic peptides and sST2, they have been found to improve either diagnosis or prognosis or both. Moving forward, rather than ignoring this important issue, studies should incorporate their use. That approach will likely lead to improved clinical outcomes for patients and particularly females. To ignore these new data will in the long run retard progress in this field.

Importantly, it is our opinion that the lack of the use of sex-specific cut off values has reduced the ability to optimally identify females at risk. It has been shown that when guidelines include appropriate strategies that take into account sex-specific differences, cardiovascular outcomes in the female population can improve [19]. However, despite better biomarker identification, it has repeatedly been shown that treatment strategies continue to be underutilized in females [20, 117]. It will take more than sex-specific cut off values to remedy the intrinsic biases that occur in cardiovascular health care [5].

Based on the information presented, we would urge that clinicians use sex-specific cut off values when they are available. The exact values will vary by analyte and clinical indication. It is only by studying these issues in appropriate populations that progress will be made in this important area. When differences are present, deploying the data therapeutically is an essential step.
